# Assessing the Quality of Use of Venous Thromboembolism (VTE) Prophylactic Devices for Stroke Patients at Leeds General Infirmary: An Audit and Re-audit

**DOI:** 10.7759/cureus.75636

**Published:** 2024-12-13

**Authors:** Daniel McWilliams, Rohid Zamani, Sameer Limaye

**Affiliations:** 1 Stroke, Leeds Teaching Hospitals National Health Service (NHS) Trust, Leeds, GBR; 2 Trauma and Orthopaedics, George Eliot Hospital, Nuneaton, GBR; 3 Surgery, Leeds Teaching Hospitals National Health Service (NHS) Trust, Leeds, GBR; 4 Surgery, University Hospitals of Coventry and Warwickshire, Coventry, GBR

**Keywords:** deep vein thrombosis (dvt), devices, geko, intermittent pneumatic compression, prophylaxis, stroke, stroke complications, venous thromboembolism

## Abstract

Background: Venous thromboembolism (VTE) is a significant cause of morbidity and mortality in patients suffering from stroke. Intermittent pneumatic compression devices (IPCs) and geko™ devices are used to reduce the risk of VTE in patients who have suffered an acute stroke. Correct use of the devices is essential for achieving the reduced risk of VTE.

Objectives: To assess the quality of use of VTE prophylactic devices by observing if they are both applied and working correctly. To identify and address factors that contribute to suboptimal use of VTE prophylactic devices.

Methods: Patients in beds 1-20 in the Acute Stroke Unit at Leeds General Infirmary were assessed on three separate days in June 2024. Data collected included: ‘VTE prophylaxis prescribed’, ‘VTE prophylaxis in place and working correctly?’, ‘If not, how so?’, ‘Notes’. Following an intervention, an identical audit was performed three months later. A total of 41 and 42 patients were included in the audit and re-audit, respectively.

Results: The audit included 35 patients who were prescribed IPCs (Kendall SCD™ 700 Smart Compression System) and demonstrated a very poor quality of use, with only 22.9% (n=35) observed to be in place and working correctly. The audit included six patients who were prescribed geko™ devices (Firstkind Ltd.) and found that 50% (n=6) were observed to be in place and working correctly. After a presentation of the results at local clinical governance and implementation of interventions, the re-audit demonstrated a substantial increase in the quality of use of both devices. The re-audit included 36 patients who were prescribed IPCs and six who were prescribed geko™ devices. The percentage of IPC and geko™ devices in place and working correctly increased to 75.0% (n=36, p<0.01) and 88.3% (n=6, p>0.01), respectively.

Conclusions: The intervention focused on increasing staff awareness of the importance of proper use of VTE prophylaxis and awareness of how to use the devices. It is reasonable to conclude that the improvement seen is a result of a change in these factors. To maintain and further improve quality, awareness of the importance of the devices and how to use them must also be maintained and improved. This should be done by additional systematic measures, such as regular training, and should be regularly reassessed.

## Introduction

Venous thromboembolism (VTE) describes deep vein thrombosis (DVT) or pulmonary embolism (PE) and is a significant cause of morbidity and mortality in hospitalized patients. Stroke patients are often at an increased risk of VTE due to immobility and multimorbidity [1-4]. Intermittent pneumatic compression devices (IPCs), also known as sequential compression devices, are designed to increase vascular blood flow and hence reduce the risk of clots by sequentially inflating a sleeve to physically compress the lower limb [5,6]. They have also been shown to stimulate endogenous fibrinolytic activity [7]. It is widely accepted that the use of IPCs lowers the risk of DVT and improves survival outcomes in stroke patients, as found in the Clots in Legs Or sTockings after Stroke 3 (CLOTS3) trial [8]. Other studies, including two large systematic reviews, found IPCs to be effective in reducing the risk of DVT in surgical patients [9,10]. One included 22 randomized control trials (RCTs) with a total of 2,779 patients and found that there was a 64% relative risk reduction in the odds of DVT (p<0.00001) [9]. 

Geko™ devices are also designed to increase vascular blood flow and hence reduce the risk of clots. They work by neuromuscular electrostimulation of the common peroneal nerve [11]. It is plausible that geko™ devices reduce the risk of VTE for stroke patients, though there is a lack of direct evidence from clinical studies to support this [12]. 

It seems clear that a high quality of use of the devices is required to achieve the reduced risk of VTE. The devices need to be consistently placed appropriately on the patients' legs and switched on to constitute a high quality of use. If the sleeves of the IPCs are not wrapped around the patients' legs or the device is often not switched on, then no benefit will be achieved. Poor compliance, equal to a poor quality of use of IPCs, is well documented in various settings [13,14]. A paper evaluating modes of failure in VTE prophylaxis commented that inadequate application of mechanical prophylaxis was common [15]. Another paper evaluating the reasons that IPCs fail to prevent DVT commented that improper use is frequent and may be the reason for failure, rather than failure of the method itself [16]. Similarly, if the geko™ devices are not appropriately sited on the patients’ legs or are not switched on, then no benefit will be achieved. Meanwhile, a cost is incurred by the department for the devices, the materials, and their use. Suboptimal use of the devices not only represents a missed opportunity to improve outcomes for patients but is also a waste of resources. For these reasons, it is essential that a high quality of use is maintained. It was noted in the Acute Stroke Unit at Leeds General Infirmary that the quality of use of prophylactic devices appeared to be suboptimal. This audit was designed to assess the quality of use of VTE prophylactic devices and identify factors that contribute to suboptimal use. 

## Materials and methods

Audit****

Aim

The aim of this audit was to identify the quality use of VTE prophylactic devices for stroke patients. 

*Objectives* 

To assess the quality of use of VTE prophylactic devices by observing if they are both applied and working correctly. To identify and address factors that contribute to suboptimal use of VTE prophylactic devices. 

*Standards/Guidelines* 

National Institute of Clinical Excellence (NICE) NG89: NICE recommends intermittent pneumatic compression for VTE prophylaxis for people who are immobile and admitted with acute stroke [6].

NICE MTG19: NICE recommends the use of the geko™ devices for use in people with a high risk of VTE when other mechanical or pharmacological methods of prophylaxis are impractical or contraindicated [17]. 

*Methods* 

Patients in beds 1-20 in the Acute Stroke Unit at Leeds General Infirmary and their electronic records were assessed between 18:00 pm and 20:00 pm on three separate days in June 2024. Data were collected on three days in the same week by one doctor familiar with the prophylactic devices. The doctor watched manufacturer instructional videos for the Kendall SCD™ 700 Smart Compression™ System (IPC) and the FirstKind Ltd. geko™ device prior to data collection. Patients who had not moved from beds 1-20 were assessed more than once over the course of the three days of data collection. Data were collected for the following: ‘NHS number’, ‘Bed number’, ‘VTE form outcome’, ‘VTE prescribed’, ‘VTE in place and working correctly?’, ‘If not, how so?’, ‘Notes’. Devices were recorded as in place correctly if they were physically present and appropriately sited on both of the patient’s legs. Devices were recorded as working correctly if the devices were switched on and working without any error messages or alarms. Patients were excluded if any of the following criteria were met: age <18, pharmacological prophylaxis prescribed, absent from the ward at the time of data collection, on end-of-life care, and no VTE prescribed. All collected data were stored and analyzed in Microsoft Excel (Microsoft Corp., Redmond, USA) and then used to perform statistical analysis with the chi-squared test [18]. 

Ethical Considerations 

The purpose of this audit was to ensure the current best practice was being met without new interventions and therefore no ethics consent was required.

*Intervention* 

The results of the audit were presented at the Stroke Department's clinical governance meeting on 17/07/2024. A multidisciplinary team (MDT) approach was taken for identifying contributing factors to the poor quality of use of devices as well as devising a strategy for improvement. Identified factors included: suboptimal staff understanding of how to use the devices, suboptimal staff understanding of the risks of VTE, failure to replace IPC devices after therapy sessions, showers, or leaving the ward for investigations, patients being unable to tolerate IPCs, and patients removing their IPCs independently. 

**Figure 1:**
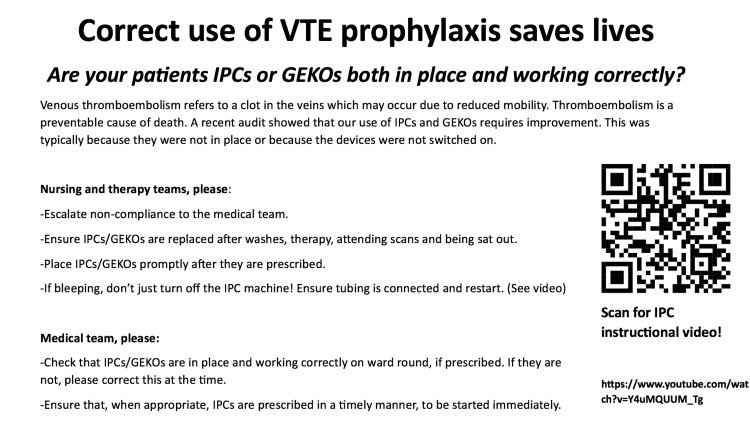
Intervention poster This poster was created to improve staff understanding of the rationale for, and practicalities of, using VTE prophylactic devices. It was displayed in several areas of the Acute Stroke Unit, and staff were directed to it at handovers. VTE: Venous thromboembolism; IPCs: Intermittent pneumatic compression devices

The poster below (Figure 1) was made following this meeting. It covers the rationale for, and practicalities of, using IPC and geko™ devices for VTE prophylaxis and includes a link to the IPC manufacturer's instructional video. It also includes specific actions for improvement for the nursing, therapy, and medical teams. A plan was made to create and share a platform for educational resources specifically for the Stroke Department that would include purpose-made training videos for VTE prophylactic devices. The poster (Figure 1) was displayed in several areas of the stroke ward. Staff were reminded of the importance of the correct use of VTE and directed to the posters at MDT handovers.

Re-audit 

Aim 

The aim of the re-audit was to assess if the intervention had influenced the quality of use of the VTE prophylactic devices. 

Methods

The methods used in this cycle were identical to those of the previous cycle. Data were collected over four days, rather than three, so that the number of patients prescribed prophylactic devices was similar. Data were collected in September 2024. 

## Results

The results of the audit and the re-audit are summarised in Table 1. The results of the audit demonstrated a very poor quality of use of IPCs with only 22.9% observed to be in place and working correctly. About 70.4% of the IPCs not being used correctly were not in place. The remaining 29.6% of the IPCs not being used correctly were appropriately sited on the patient’s legs but not working correctly. In seven of the eight cases, this was because the device was not switched on. In the remaining case, there was a loose connection message showing on the device. The use of geko™ devices was of higher quality; however, they were still suboptimal, with only 66.7% observed to be in place and working correctly. 

**Table 1:** Summary of audit versus re-audit findings The table summarises the data from Cycle 1 and Cycle 2, showing the number of devices that were in place and working correctly for IPC and geko™ devices. It shows the breakdown of devices that were not in place versus not working correctly as a percentage of the devices that were not in place and working correctly. The change in the number of IPCs and geko™ devices that were in place and working correctly was assessed for statistical significance using the chi-squared test. IPCs: Intermittent pneumatic compression devices

	Cycle 1			Cycle 2			
	Number of devices	Total number of devices assessed	Percentage	Number of devices	Total number of devices assessed	Percentage	P-value for the change in the number of devices
IPCs in place and working correctly	8	35	22.9	27	36	75.0	<0.01
IPCs that were not in place	19	27	70.4	5	9	55.6	Not applicable
IPCs that were not working correctly (i.e., in place but not switched on or an error code)	8	27	29.6	4	9	44.4	Not applicable
Geko™ devices in place and working correctly	3	6	50.0	5	6	83.3	>0.01
Geko™ devices that were not in place	2	3	66.7	0	1	0.0	Not applicable
Geko™ devices that were not working correctly (i.e., in place but not switched on)	1	3	33.3	1	1	100.0	Not applicable

The results of the re-audit showed a significant improvement in the quality of use of prophylactic devices, with an increase in IPCs both in place and working correctly from 22.9% to 75.0%. There was an increase in geko™ devices both in place and working correctly from 50.0% to 83.3%. Of the IPCs observed to not be in place and working correctly, there was an almost even proportion of devices not in place versus not working correctly. 

The statistical level of significance between the number of IPCs in place and working correctly observed in the audit versus the re-audit was assessed using the chi-squared test with a critical value corresponding to a p-value of 0.01. The increase in the number of IPCs in place and working correctly was found to be statistically significant (p<0.01). The statistical level of significance between the number of geko™ devices in place and working correctly observed in the audit versus the re-audit was also assessed using the chi-squared test with a critical value corresponding to a p-value of 0.01. The increase in the number of geko™ devices in place and working correctly was found not to be statistically significant (p>0.01). 

## Discussion

The Acute Stroke Unit can often be a busy place with significant demands on all staff. A range of professionals are involved in the MDT approach, which is essential to cater to the complex needs of stroke patients. In an environment of such acuity where collaborative working is necessary, attention to fine details may be missed or even viewed as a low priority. In the context of the quality of use of VTE prophylactic devices, this may mean a delay in the application of devices from the time of prescription, failing to regularly check that devices are sited and working appropriately, failing to replace IPCs after being out of bed or silencing an alarm from an IPC device, perhaps by switching it off, and failing to address the issue at a convenient time. In a fast-paced and dynamic environment, ensuring the quality of use of devices is challenging. Careful attention to the quality of use of VTE prophylactic devices may be overlooked. However, these details are important for patient outcomes. 

The CLOTS3 trial was a large RCT involving 2,872 stroke patients, which found a statistically significant improvement in survival to six months for those treated with IPCs versus those who were not [8]. The trial found that there was an adjusted odds ratio of 0.65 (95% CI 0.51 to 0.84; p=0.001) for the occurrence of DVT in patients treated with IPCs versus those who were not. It concluded that the use of IPCs was an effective and inexpensive method of reducing the risk of DVT and improving survival among immobile stroke patients [8]. A further paper assessing the same data found that after six months, there were no significant differences in disability, institutional versus non-institutional living circumstances, or health-related quality of life between patients treated with IPCs versus those who were not [19]. The same paper estimated that the cost of treatment with IPCs to be £64.10 per patient (SD 28.3). Additionally, a systematic review and meta-analyses including 3,551 stroke patients found a risk ratio of 0.50 for the occurrence of DVT in those treated with IPCs even though there was no statistically significant increase in quality-adjusted survival. It also found an increase in IPC-associated adverse events [20]. 

There is very little available literature specifically assessing the quality of use of, or compliance with, VTE prophylactic devices for stroke patients. There is no clear guidance on the required length of use per day to achieve the reduced risk of VTE. In the CLOTS3 trial, IPCs were worn day and night for at least 12 days [8]. The CLOTS3 trial provides the most robust evidence for the use of IPCs for stroke patients and hence best practice is based on this trial. Therefore, IPCs should be worn as much as possible day and night to meet the best practice standard of care. This audit and re-audit observed the quality of use of IPCs to assess compliance with this standard, in accordance with NICE guideline NG89 [6]. Compliance with the best practice can be assumed based on the quality of use observed. For example, a very high observed quality of use would suggest that the IPCs are almost always in place and working correctly, thus meeting the best practice standard. 

The results of this audit found a very poor quality of use of IPCs, with only 22.9% of devices observed to be in place and working correctly. This suggests that the length of time per day that IPCs were worn in an effective way was significantly lower than the best practice. The benefits of improved survival outcomes and decreased risk of DVT would not be achieved with this very poor quality of use of IPCs. It is likely that preventable VTE would occur with the quality of use of IPCs seen in this audit. The results of the re-audit found a statistically significant increase in the number of IPCs in place and working correctly (p<0.01), proving the short-term success of the intervention. Whilst the re-audit demonstrated an achievement with a significant increase to 75% of IPCs in place and working correctly, there remains the need for improvement. The increase in the number of geko™ devices in place and working correctly was not statistically significant (p>0.01). This was likely due to the small number of devices assessed, which was a total of six in the audit and six in the re-audit.

The results of this audit are comparable with those found in a project to improve compliance with IPC treatment in trauma patients in Kings County Hospital Center, New York. A baseline audit found a compliance rate of 45% [13]. The reasons identified for poor compliance included: uncomfortable sleeves (62%), sleeves removed before bedtime (15%), sleeves removed after leaving bed (13%), and IPC machine not switched on (6%). The intervention focussed on patient education. Similar to the findings of our audit, the most common reason for poor quality of use was IPCs not being placed correctly. Another project that aimed to improve compliance with IPC treatment found that the baseline compliance in neurosurgical patients was 19.7% (n=95) and 38.4% (n=131) for two consecutive months prior to intervention [21]. This project found that nursing-focused intervention facilitated sustained improvement though not beyond 50% compliance. Further improvement was seen following a patient-focussed intervention. Whilst patient-focused intervention may not be appropriate for a proportion of stroke patients due to the nature of their condition, the success seen in these projects presents an interesting opportunity for further intervention to improve the quality of use of prophylactic devices for stroke patients.

This audit and re-audit have limitations to consider. Data was collected at only one moment in time per day per patient. There was an assumption that the quality of use observed at this time was representative of the average quality of use for the day. Furthermore, no data was collected on a weekend where factors such as staffing levels may have an effect on the quality of use of VTE devices. In addition, data was collected from the Acute Stroke Unit only. The Stroke Department had a significant number of outlying patients in alternative wards for whom the quality of use of VTE devices was not assessed. It would seem logical to assume that in these wards, where IPCs and geko™ devices may not be as commonly used, the quality of use would be lower. 

The intervention focused on increasing staff awareness of the importance of proper use of VTE prophylaxis and awareness of how to use the devices. It is reasonable to conclude that the improvement seen is because of a change in these factors. To maintain and further improve quality, awareness of the importance of, and how to use, the devices must also be maintained and improved. This should be done through additional systematic measures such as regular training and improving educational resources. Regular reassessment will ensure maintained quality and prompt any further interventions that may be required. The Stroke Department has placed such high importance on this subject that it has made this audit a mandatory internal audit every six months. 

## Conclusions

This audit assessed the quality of use of prophylactic devices in the Acute Stroke Unit at Leeds General Infirmary, West Yorkshire, UK, and demonstrated a very poor quality of use of IPCs by finding that only 22.9% were both appropriately sited and working correctly over three days in June 2024. The audit results were presented at local clinical governance and action was taken to raise awareness of the importance of proper use of VTE prophylaxis and awareness of how to use VTE devices. A re-audit three months later demonstrated a statistically significant improvement in the quality of use of IPCs, with 75% of IPCs in place and working correctly. This means that the best practice standard for the quality of use of IPCs is closer to being met. The Stroke Department has placed high importance on this subject and made it a mandatory internal audit every six months. 
